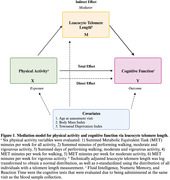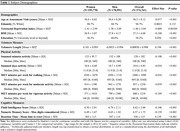# Sex differences in the relationship between physical activity and cognition as mediated by leukocyte telomere length: a UK Biobank study

**DOI:** 10.1002/alz.091083

**Published:** 2025-01-03

**Authors:** Nancy E Ortega, Vahan Aslanyan, Judy Pa

**Affiliations:** ^1^ Alzheimer’s Disease Cooperative Study (ADCS), University of California San Diego, La Jolla, CA, USA, La Jolla, CA USA; ^2^ Neurosciences Graduate Program, University of California San Diego, La Jolla, CA, USA, La Jolla, CA USA; ^3^ Department of Population and Public Health Sciences, Keck School of Medicine, University of Southern California, Los Angeles, CA USA; ^4^ Alzheimer’s Disease Cooperative Study (ADCS), University of California, San Diego, La Jolla, CA USA; ^5^ Neurosciences Graduate Program, University of California, San Diego, La Jolla, CA USA

## Abstract

**Background:**

Evaluating sex differences in modifiable risk factors for Alzheimer’s disease could provide valuable information regarding the mechanisms by which these factors confer risk. Physical activity is a risk factor that has been shown to positively impact both telomere length, a marker of cellular age, and cognition. The goal of this study was to evaluate whether telomere length mediates the association between physical activity and cognition differently by sex.

**Method:**

Participants from the UK Biobank were included in the sample based on availability of demographic variables, physical activity questionnaires, leukocyte telomere length, and cognitive measurements (Fluid Intelligence (FI), Numeric Memory (NM), Reaction Time (RT)). Metabolic Equivalent Task (MET) scores were derived from self‐report questionnaires following the International Physical Activity Questionnaire (IPAQ) guidelines. Relative leukocyte telomere length was measured by quantitative PCR and adjusted for technical variation by the UK Biobank. The indirect effect of physical activity on cognition via telomere length was evaluated using causal mediation analyses in R. Models were adjusted for age, body mass index, and Townsend Deprivation Index (Figure 1). A priori sex‐stratified models were used to examine sex differences.

**Result:**

The sample included 374,761 adults (52.2% women, mean age±SD = 56.3±8.12 years, see Table 1). Linear regression analyses identified negative associations between weekly physical activity measures (i.e., summed MET minutes of all activity p<0.05*, MET minutes of moderate activity p<0.001***) and telomere length in men. This negative relationship was similarly found in women (MET minutes of walking p<0.05*), except for MET minutes of vigorous activity where a positive relationship with telomere length was observed (p<0.05*). In men, telomere length mediated the relationship between MET minutes of moderate activity and FI scores (p<0.001***), whereas in women this was only seen for walking (p = 0.04*). The association between vigorous activity and performance on the NM task was mediated by telomere length in women only (p = 0.04*). Lastly, telomere length mediated the relationship between RT performance and summed minutes of all activity in men only (p<0.001***).

**Conclusion:**

Telomere length mediates the relationship between physical activity and cognition differently in men and women based on the intensity level of the physical activity.